# Q sample construction: a novel approach incorporating a Delphi technique to explore opinions about codeine dependence

**DOI:** 10.1186/s12874-019-0741-9

**Published:** 2019-05-14

**Authors:** Melissa Kirschbaum, Tony Barnett, Merylin Cross

**Affiliations:** University of Tasmania Centre for Rural Health, Locked Bag 1372, Launceston, Tasmania 7250 Australia

**Keywords:** Attitudes, Codeine, COM-B, Delphi technique, Drug addiction, Q methodology, Mixed methods

## Abstract

**Background:**

Q methodology is an evidenced approach to researching subjectivity, involving a combination of qualitative and quantitative techniques. The methodology has been used successfully in healthcare research to explore the opinions of patients and healthcare providers about topics such as the illness experience, healthcare services, clinical practice and professional training. Q methodology studies require the generation of a Q sample, a set of opinion statements representing the phenomenon of interest. This paper describes a novel and rigorous approach to develop a Q sample for a study exploring misusers’ opinions about over-the-counter (OTC) codeine dependence and critically examines the associated methodological issues.

**Methods:**

Development of the Q sample in this study involved three steps; (1) identification of opinion statements via a comprehensive literature search, (2) application of a theoretical framework, the Capability, Opportunity, Motivation - Behaviour (COM-B) model of behaviour, to group and then reduce the number of statements and (3) use of a Delphi technique to achieve expert consensus on the final selection of statements. The Delphi component involved a multidisciplinary panel of 15 addiction experts comprised of doctors, nurses, pharmacists, psychologists and researchers, who were recruited purposively. Experts rated each statement using a 5-point scale of perceived importance. Two Delphi rounds were undertaken and consensus for inclusion of a statement was set at a median score of ≥4 and an interquartile range of ≤1.

**Results:**

A total of 842 statements representing codeine misusers’ opinions about OTC codeine dependence were identified from the literature. Statements were grouped thematically using the COM-B framework and representative statements were selected, reducing the number to 111. After two Delphi rounds, addiction experts achieved consensus on 46 statements which formed the final Q sample.

**Conclusions:**

This paper describes a new and systematic approach to Q sample construction and explores associated methodological issues that could be useful for those considering Q methodology and for furthering the rigour of this research technique.

## Background

Attitudes about health and healthcare guide health behaviours and shape peoples’ healthcare experience. An understanding of these beliefs is therefore vital for the effective design, delivery, evaluation and optimisation of health services. Q methodology provides an evidenced approach to research subjective understanding. It has been successfully used in healthcare research to explore the views of patients, the general public, healthcare providers and stakeholders about a diverse range of topics including the illness experience [1], patient decision-making [2], quality of healthcare [3, 4], clinical practice [5, 6], health policy [7, 8], health economics [9] and professional training [10, 11].

Q methodology was developed by English psychologist and physicist, William Stephenson [12, 13] as a method to explore human subjectivity. It is as an adaptation, or inversion, of traditional R methodological factor analysis. The term ‘Q’ was chosen to distinguish it from ‘R’ methodology, with the ‘R’ relating to Pearson’s r [14]. Stephenson contended that R-methodological factor analysis, with its focus on general population level comparisons of tests or traits, could not provide a holistic representation of the differences between individuals. A solution was to consider people rather than tests or traits as variables, with by-person factor analysis used to reveal factors representing “persons who resemble one another with respect to whole aspects of their personality” [15].

Q methodology involves a combination of qualitative and quantitative techniques. For the quantitative component, participants rank statements representing existing opinions on the research topic according to their personal views. Typically, the statements are arranged by participants from ‘most disagree’ to ‘most agree’, in a fixed normal distribution grid. Participants are treated as variables across the sample of statements and by-person analysis is used to identify factors that represent common ways of thinking. The factors are then interpreted in a qualitative manner, often with the aid of supporting data collected during post-sort participant interviews [16].

A Q methodological study commences with the generation of the concourse, a comprehensive set of opinion statements that represent the phenomenon of interest. Concourse statements can be derived in numerous ways, such as review of the literature (including scholarly literature and popular media), established attitude scales, interviews or focus groups with potential study participants, and personal experience of the researcher [17]. A sample of statements, known as the Q sample, is then selected from the concourse to represent the key concepts and ideas associated with the research questions and phenomenon under investigation.

The most formal Q sample structure, as favoured by Stephenson, is based on Fisher’s variance design [14], in which the topic is conceptualized theoretically using a matrix structure and equal numbers of statements are selected from each matrix cell. For example, Brown [14] in a study that explored meanings of “being in love”, proposed that statements could be conceptualized as either romantic or realistic. These could then be categorized further as relating to either the self or interactions with others. Statements could then be drawn from each of the four resulting matrix cells: romantic-self, romantic-interaction, realistic-self and realistic-interaction. In contrast, an unstructured sample affords the researcher more flexibility and creativity as the topic is considered as a whole, rather than being subdivided into parts [16]. Whilst different approaches may be used, the goal is to generate a Q sample of statements that is manageable for participants to work with and that is broadly representative of the concourse. Typically, the size of the Q sample ranges between 40 and 80 statements, although there is little evidence, other than precedence, to justify this recommendation [16].

Q methodology has been criticized for lack of transparency and detail around Q sample construction. For example, it has been stated that “the QM (Q methodology) literature remains uncomfortably silent with respect to how to assemble and verify completeness of a concourse, and how to verify or falsify the representativeness of a sample drawn therefrom” [18]. It has also been claimed that “within the Stephenson tradition…. a set of Q items typically is quickly assembled, structured a priori (often questionably) by the investigator…, and is not itself further evaluated as to its sufficiency of meaning” [19]. Critics have also questioned the influence of researcher bias in the process of Q sample construction, including when choosing the population from which to derive the concourse, selecting concourse statements and sampling the concourse to form the Q sample [18].

In response, more recent reports have attempted to address these shortcomings and criticism [20–22]. This paper extends this work by making explicit, the procedures used for the development of an exemplar Q sample. The Q sample is drawn from a study that explored over-the-counter (OTC) codeine misusers’ opinions about their dependence. The Q sample was designed to represent opinions about OTC codeine dependence from the perspective of the misuser. Codeine misusers were therefore recruited to undertake the Q sort process. Methodological issues encountered during the study are identified and discussed. These included strategies to: reduce researcher bias; generate a comprehensive concourse; select the Q sample (size and representation, use of a theoretical framework); constitute a Delphi panel (size and membership); define consensus; and resolve language issues.

Construction of this Q sample included the use of a Delphi technique to facilitate expert consensus for statement selection. Although it is common for statements to be chosen by the researcher [14, 16], in this study, expert consensus was used to reduce researcher bias. The Delphi technique has been used previously in combination with Q methodology to generate a concourse [23], pilot a Q sample [23] and to explore the subjectivity behind decision making in each Delphi round [24]. To our knowledge, this is the first application utilizing the Delphi technique for the purpose of statement selection.

This paper describes a novel and rigorous approach used to develop a Q sample for a study exploring opinions about OTC codeine dependence, the issues arising and strengths and limitations of the process.

## Methods

**Fig. 1:**
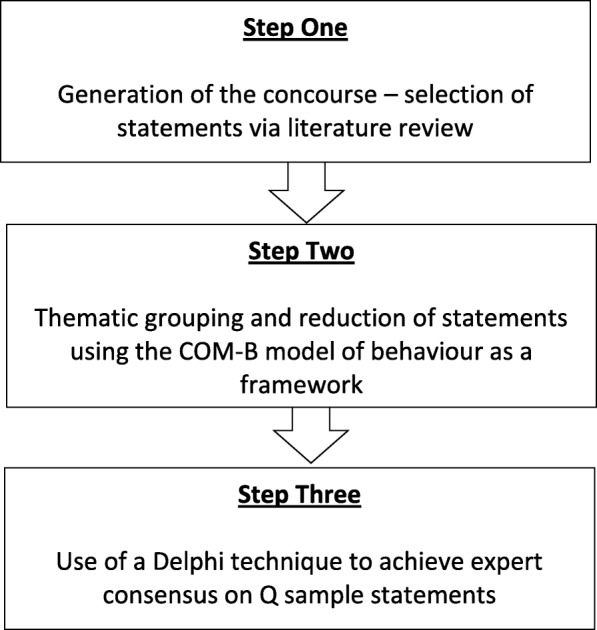
Method of Q sample construction

Construction of this Q sample involved three steps (Fig. 1), each of which was designed to reduce the influence of researcher bias. The first step, generation of the concourse, involved identification of opinion statements via a comprehensive literature search. This ensured a wide population from which to draw the concourse. For the second step, an established model, the Capability, Opportunity, Motivation-Behaviour (COM-B) system, was used as a framework to guide decision-making when grouping and then reducing statements. Lastly, a Delphi technique was used to achieve expert consensus on the final selection of statements.

### Step 1. Generation of the concourse

Concourse statements were identified through a review of scholarly and grey literature, including websites, public submissions and online discussion forums, undertaken between October 2016 and February 2017. Details of the search strategy are shown in Table 1.

**Table 1:** Search strategy used to generate the concourse

Document type	Source	Search string and limits
Scholarly literature	CINAHL, Google Scholar, Proquest, Pubmed, Scopus, Web of Science	Codeine AND (over-the-counter OR OTC OR non-prescription) AND (addiction OR dependence OR abuse OR misuse), limited to 2006-October 2016. Q methodolog* AND (addiction OR dependence OR abuse OR misuse), limited to 2006-January 2017
Validated addiction instruments	Substance Use Screening and Assessment Instruments Database	Full manual screen by title
Theses	Trove, Bielefeld Academic Search Engine, EThOs, Proquest Dissertations and Theses	OTC codeine OR over-the-counter codeine OR over the counter codeine OR non-prescription codeine OR nurofen plus OR panadeine OR mersyndol OR dolased
Newspaper, magazine, radio	Factiva	(OTC codeine OR over-the-counter codeine OR over the counter codeine OR non-prescription codeine OR nurofen plus OR panadeine OR mersyndol OR dolased) AND (addiction OR dependence OR abuse OR misuse), limited to 2006-February 2017
Websites of professional organizations - pharmacy, addiction	Google	Manual identification of specialist addiction research centres and pharmacy and addiction professional associations
Reports	Google	(OTC codeine OR over-the-counter codeine OR over the counter codeine OR non-prescription codeine) AND report, ordered by relevance and limited to the top 20
Public submissions	www.tga.gov.au/scheduling-submission/public-submissions-scheduling-matters-referred-acms-15-august-2015	Full manual screen, all submissions read and reviewed in full
Online forums	Google	(OTC codeine OR over-the-counter codeine OR over the counter codeine OR non-prescription codeine OR nurofen plus OR panadeine OR mersyndol OR dolased) AND (addiction OR dependence OR abuse OR misuse) AND (forum OR blog OR thread OR post), ordered by relevance and limited to the top 20

Statements that represented opinions held by OTC codeine misusers about their misuse, including the causes of addiction, reasons for drug use, locus of control, identity, harms, consequences, treatment and prevention strategies were extracted. Records were screened for relevance by title and abstract. Articles were also identified from the reference lists of included papers and key researchers. In reviewing relevant Q methodology research papers and validated addiction instruments, statements about drug misusers generalizable to OTC codeine misusers were also considered for selection. Collection of statements was ceased when the search strategy had been fully executed and it was found that no new statements had emerged, that is data saturation was reached.

### Step 2. Thematic grouping and reduction of statements using the COM-B framework

The COM-B model of behaviour [25] was developed by Michie et al. in 2011 as a comprehensive model for understanding behaviour, based on existing behavioural theories. The model proposes that behaviour is a result of the interaction between Capability (physical and psychological), Opportunity (physical and social) and Motivation (reflective and autonomic). The COM-B forms the centre of the Behaviour Change Wheel [25], where it is encircled by intervention strategies and then policy options to facilitate behaviour change. The Behaviour Change Wheel, initially applied to tobacco control and obesity reduction [25], has subsequently been used in a variety of healthcare contexts [26–28].

The COM-B has been used in addiction research as an overarching model to integrate concepts from multiple theories of addiction [29]. It was therefore considered suitable to inform the development of the Q sample in the current study. The three COM-B domains, as well as the headings used by West in his application of the COM-B model to addiction research [29], formed the Q sample structure (see Table 2).

**Table 2:** The COM-B model of behaviour applied to addiction research

Capability	Knowledge of and ability to understand consequences. Self-regulatory capacity and skills. Knowledge of and ability to understand how to change.
Opportunity	Access to the addictive behaviour. Cues in the physical and social environment that prompt or remind about the addictive behaviour. Cues in the social environment that would permit or prompt change.
Motivation	Beliefs about the positive and negative features of the addictive behaviour. Pleasure and satisfaction derived from the addictive behaviour. Mental and physical discomfort arising from the addictive behaviour. Needs met by the addictive behaviour. Pleasure and satisfaction derived from, and needs met by, other activities. General aspects of identity. Aspects of identity relating to the addictive behaviour.

Source: adapted from West R. Models of Addiction. EMCDDA Insight Series NO14. Luxembourg: European Monitoring Centre for Drugs and Drug Addiction; 2013. p. 111–2

The opinion statements identified in Step 1 were sorted thematically using the COM-B framework (Table 2). Statements were assigned to one thematic group only. The number of statements were then reduced by selecting representative statements from each thematic group. Preference was given to statements that demonstrated; (1) content from sources specifically describing OTC codeine dependence or from validated addiction instruments, (2) simple language suitable for lay people, (3) use of personal pronouns, and (4) relevance to the Australian context. Duplicated statements and those which represented the same meaning though worded slightly differently were removed. Where an opinion statement was represented by both a positive and negatively worded form, the more readable (simpler) statement was selected. Residual statements were then reworded where necessary for clarity, to simplify the language, to describe one issue only (not double-barrelled), to use personal pronouns, to make specific for OTC codeine dependence and to be relevant for people both aware and unaware of their dependence.

### Step 3. Use of a Delphi technique to select the Q sample

The Delphi technique is a structured method to facilitate consensus of expert opinion. Although initially developed for military forecasting [30], it has since been applied to many research areas including healthcare [31]. The technique involves a panel of experts who undertake a series of questionnaire rounds. Panel member anonymity is maintained, reducing the influence of dominant personalities on the decision-making process. After each Delphi round, feedback is given about the group opinion. In the subsequent round panel members are given the opportunity to revise their individual responses in light of this feedback. This iterative process continues until consensus is achieved [32].

For this study, a two-round Delphi technique was used to further reduce the number of statements and to achieve expert consensus on the statements to include in the Q sample. The Delphi component was conducted from June to August 2017. Ethical approval was provided by the Social Sciences Human Research Ethics Committee of the University of Tasmania.

### Panel selection

An international multidisciplinary team of addiction experts were recruited purposively and via snowball sampling. Experts were defined as researchers or clinical professionals (doctors, nurses, pharmacists, psychologists) with at least 2 years’ experience in the field of addiction (including OTC codeine addiction).

### Round one

Twenty-five identified addiction experts were emailed invitations to participate in June and July 2017. The invitation provided information about the purpose of the study, including an explanation that the presented statements reflected the opinions of OTC codeine misusers and were drawn from the literature. The instruction given to each panel member was to rate their agreement on statements that would allow OTC codeine misusers to express their views about their dependence. In rating each statement, the experts were requested to consider the relevance of each statement based on their experience with OTC codeine misusers, their knowledge of the theories of addiction and that the overall intention was to reduce the number of statements. Experts were advised that participation involved a commitment to multiple rounds. A reminder email was sent seven to ten days after the initial invitation if no response was received.

The Round One online survey, administered using Lime Survey [33], was accessible via a direct hyperlink from the invitation email. Initial questions focussed on the collection of socio-demographic information. Experts were then asked to rate 111 statements, sequenced according to COM-B thematic groupings, on a 5-point Likert scale on how important they felt it was to include each statement in the Q sample (1 = not important, 5 = very important). They were also asked to indicate which statements, if any, required rewording and to nominate additional statements if they considered something important was missing.

Responses from panel members to each statement were entered onto a spreadsheet. Entries were checked and descriptive statistics computed for each statement, including the median and interquartile range (IQR). An IQR ≤ 1 was chosen to indicate consensus amongst panel members, as this has been suggested as a good indicator of consensus for 5-point Likert scales [34–39]. Statements were included in the Q sample if there was expert consensus (IQR ≤ 1) and if the statement was rated as important (indicated by a median score of ≥4).

### Round two

Round Two was undertaken in August 2017. At the commencement of the round, panel members were informed that, based on feedback, statements would be reworded where necessary to replace ‘addict’ and ‘addiction’ with ‘dependence’ once the statement list had been finalised. This change in terminology was undertaken to help reduce possible stigmatisation of participants.

Statements that achieved the median requirement (≥4), but for which panel members did not reach consensus (IQR > 1), were presented again to the panel in Round Two. Any new statements suggested by the panel in Round One were also presented for rating. Each panel member was emailed a hyperlink directing them to their own unique Lime Survey, containing their individual Round One response, the median group response and IQR for each statement. The experts were invited to re-rate the statements considering this feedback using the same 5-point Likert scale used in Round One. Non-responders received up to two reminder emails.

Median and IQRs were again calculated for each statement to identify those statements that the experts agreed were important to include (median score of ≥4 and IQR ≤ 1). The statements that achieved consensus for inclusion in the Q sample were reworded if necessary in light of panel member feedback and were placed back into the COM-B framework to check for coverage of themes.

## Results

### Step 1. Generation of the concourse

A total of 842 statements were extracted from the literature search to form the concourse (Table 3).

**Table 3:** Results for the generation of the concourse and Q sample

Search results	Document type							
	Scholarly literature	Validated addiction instruments	Theses	Newspaper, magazine, radio	Websites of professional organizations - pharmacy, addiction	Reports	Public submissions	Online forums
Number of records identified for screening	156 codeine + 25 Q methodology articles after duplicates removed	980 instruments	22 theses after duplicates removed	541 articles after duplicates removed	18 websites	53,600 with top 20 screened	236 submissions	691,000 hits with top 20 screened
Number of records from which statements were extracted for concourse	22 codeine + 4 Q methodology articles + 2 articles identified via snowball sampling and professional organization websites	6 instruments + 1 identified via snowball sampling	1 thesis (also previously identified in scholarly literature search)	21 articles + 1 identified via professional organization website	1 website (led to the extraction of statements from linked journal and magazine articles – included under journal and magazine headings)	2 reports (including one already identified in newspaper search) + 1 report identified via snowball sampling	55 submissions	12 forums
Number of statements extracted for concourse (842)	202 (+ 6 included under thesis heading)	208	6	28	0	150 (+ 1 included under newspaper heading)	111	137
Number of statements included in initial reduction using COM-B (111)	17	30	1	7	0	20	12	24
Number of statements included in Q sample (45 + 1 added by Delphi panel)	7	10	0	4	0	8	7	9

### Step 2. Thematic grouping and reduction of statements using the COM-B framework

The 842 statements were sorted thematically using the COM-B domains and headings (Table 2). Representative statements were then selected, resulting in 111 residual statements covering each of the COM-B addiction headings, with the exception of “General aspects of identity” and “Pleasure and satisfaction derived from, and needs met by, other activities”. “General aspects of identity” was not represented in the initial concourse. It is, however, closely related to “Aspects of identity related to the addictive behaviour”, therefore statements about identity were felt to be reasonably represented. “Pleasure and satisfaction derived from, and needs met by, other activities” only contained two statements, neither of which were specific to OTC codeine, therefore this heading was not considered critical to represent in the context of this study.

### Step 3. Use of a Delphi technique to select the Q sample

#### Participants

Fifteen experts completed the Round One online survey. This included doctors (*n* = 3), nurses (n = 3), pharmacists (*n* = 4), academic pharmacists (*n* = 2), a psychologist and researchers (n = 2). Most worked in Australia (*n* = 9), though other countries were also represented: Ireland (*n* = 1), New Zealand (n = 1), Singapore (n = 3) and the United Kingdom (n = 1). Thirteen experts were recruited by direct invitation by the research team and two via participant referral. The average age of panel members was 47 years (range 30–59) and eight (53%) were female. The panel retention rate for Round Two was 73.3%, with eleven of the experts completing the survey. Non-responders for Round Two included two doctors, one nurse and one pharmacist.

#### Round one

**Fig. 2:**
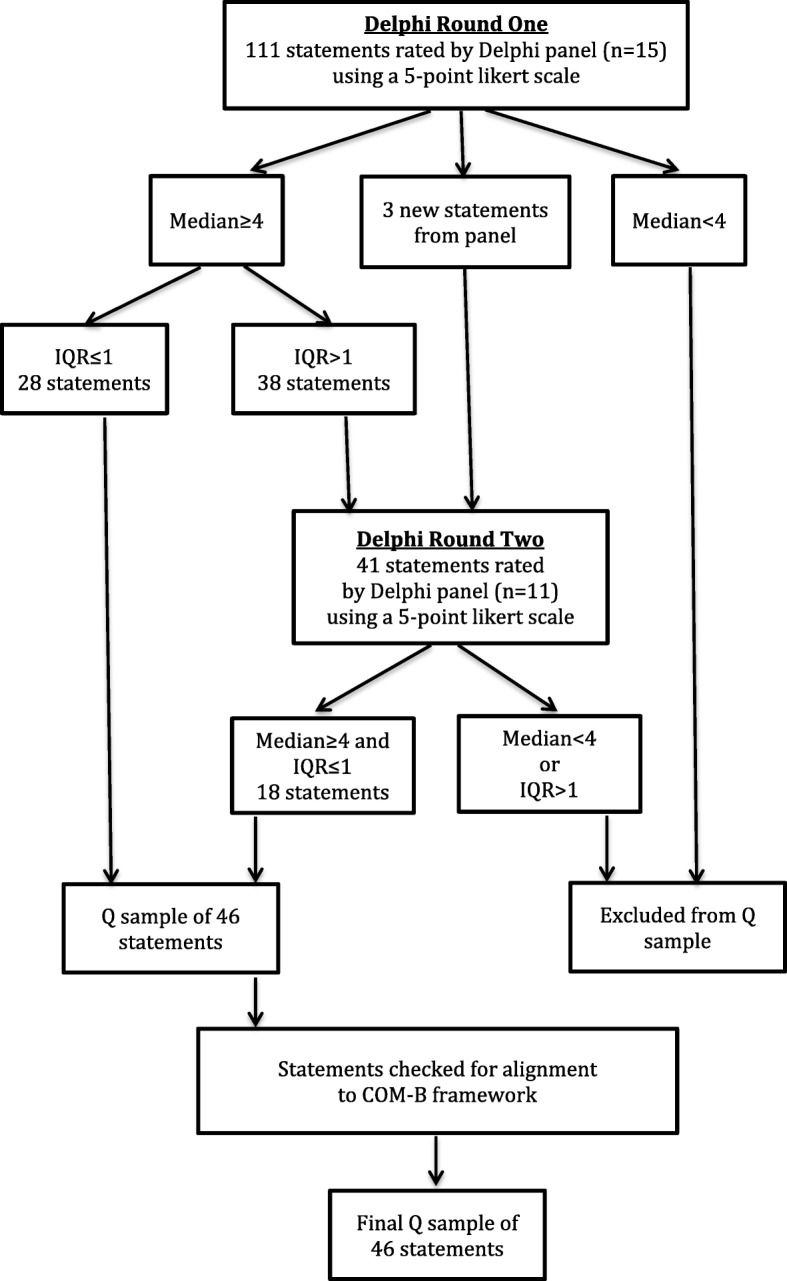
Results of the two-round Delphi survey

Twenty-eight statements achieved consensus for inclusion (median score of ≥4 and IQR ≤ 1) in Round One (see Fig. 2). The average time taken to complete Round One was 29 min. Two of the fifteen experts expressed concern over the use of the terms ‘addiction’ and ‘addict’, and instead suggested the use of ‘dependence’ or ‘substance use’: “hopefully this will reduce stigmatising those involved in the study… through avoiding terminology such as ‘addicts’” (Participant 2) and “I would suggest the word addict not be used - nor drug addiction, substance use is a preferred way of thinking about this” (Participant 3).

#### Round two

The 38 statements that achieved the median requirement (≥4), but for which panel member responses were more widely spread (IQR > 1) in Round One were presented again to the panel in Round Two. In addition, three new statements suggested by panel members were included: “I use codeine to help relieve stress” (Participant 2); “I am aware of the damage that codeine does to my internal organs” (Participant 7) and; “I use codeine as an alternative to heroin” (Participant 13). The average time taken to complete this round was eight minutes. Eighteen statements achieved consensus for inclusion during Round Two (see Fig. 2), including one of the statements suggested by the panel; “I use codeine to help relieve stress”.

Over the two Delphi rounds, the experts agreed on 46 statements to include in the Q sample; 28 statements were generated from Round One and 18 statements from Round Two. No further rounds were conducted as the Q sample size fell within the desired range of 40–80 statements and all four COM-B domains were represented.

The 46 statements were reworded where necessary to replace the term addiction with dependence in response to panel feedback. For example, “it is not an addict’s fault that they are addicted” became “it’s not a person’s fault if they become dependent on OTC codeine”. The statements were again mapped against the COM-B model (Table 2) to ensure the major domains: Capability, Opportunity and Motivation were represented. Two of the headings (in addition to the two headings previously not represented), “Access to the addictive behaviour” and “Cues in the social environment that would permit or prompt change”, were not specifically represented, however due to overlap of thematic content they were considered to be broadly represented. The final Q sample statements, organized into the broad COM-B domains, are listed in Table 4.

**Table 4:** Q sample statements (*n* = 46) mapped to the COM-B domains

Capability (*n* = 17)	
I am fully aware that I am consuming more OTC codeine than is recommended. I ignore the dangers of regularly using OTC codeine. When I first started taking OTC codeine, I didn’t even know that you could become dependent on it. Since OTC codeine is available without a prescription it must be safe. It’s not the codeine that is the problem in the tablets, it’s the paracetamol and ibuprofen. I ignore the directions on the OTC codeine box. I take OTC codeine out of habit, more than for any other reason. Recovery from drug dependence is a continuous process that never ends. I think OTC codeine dependence is connected with having an addictive personality. It’s not a person’s fault if they become dependent on OTC codeine. A person dependent on OTC codeine needs professional help. Treatment centres are only provided for addictions that are considered more serious than OTC codeine dependence. There is nowhere that people dependent on OTC codeine can go for help. Health professionals are very dismissive when it comes to OTC codeine dependence. Health professionals have little knowledge about OTC codeine dependence. People dependent on OTC codeine should not have to receive treatment together with people dependent on other types of drugs. Medication is helpful in supporting recovery from OTC codeine dependence.	
Opportunity (*n* = 5)	
Making codeine prescription-only denies patients the right to timely pain relief. Recording OTC codeine buyers’ names on a national database is a suitable solution to the problem of OTC codeine dependence. Taking OTC codeine on a regular basis is socially acceptable. I use OTC codeine to overcome personal problems. I use OTC codeine because circumstances force me to do so.	
Motivation (*n* = 24)	
OTC codeine can lead people to use even stronger drugs. Daily use of OTC codeine is not necessarily harmful. The dangers associated with the use of OTC codeine are exaggerated. OTC codeine dependence isn’t recognised as a serious problem. OTC codeine misuse is a big problem in the community. Anyone can become dependent on OTC codeine. You can’t tell that a person is dependent on OTC codeine by looking at them. People who take OTC codeine become dependent by accident. There is little difference between an OTC codeine addict and an injecting drug addict. My life on OTC codeine is better than life without it. To stop taking OTC codeine would be like losing part of myself. I am a better person without OTC codeine. Stopping OTC codeine would be like losing a best friend. Being seen as a regular user of OTC codeine doesn’t bother me. I use OTC codeine as a way to relax. I hide my use of OTC codeine from others. I feel ashamed of using OTC codeine. I always regret taking OTC codeine. I give a lot of thought to what OTC codeine is doing to my health. I think less of myself because I use OTC codeine. I take OTC codeine to treat physical pain. I take OTC codeine to cope with life. I have to take OTC codeine to feel normal. I use OTC codeine to help relieve stress.	

## Discussion

This manuscript has explicitly described a new approach to constructing a Q sample. Methodological issues that arose during the process are now discussed, including strategies to: reduce researcher bias; generate a comprehensive concourse; select the Q sample (size and representation, use of a theoretical framework); constitute a Delphi panel (size and membership); define consensus; and resolve language issues.

### Reduction of researcher bias

The potential for researcher bias has been acknowledged in both quantitative and qualitative research and various strategies have been suggested to mitigate this risk [40]. Likewise, researcher bias has been identified as a significant challenge in the process of Q sample construction, with critics suggesting that “if reflexivity is not adequately considered, Q sorting has the inherent risk of turning into a Socratic dialogue, wherein Socrates (the researcher) with great certainty obtains the correct responses from Trasymachus (the respondent)” [18]. In other words, researcher bias may result in the selection of statements that solely represent the view that the researcher expects or seeks to find and could therefore produce mis-leading results. The combination of the three steps used to construct the Q sample in this study was specifically designed to reduce this risk.

### Comprehensiveness of the concourse

Although it is common for concourse statements to be derived from existing literature [16], few studies describe in detail an extensive review process involving a wide range of sources. For the current study, a comprehensive review of the literature was undertaken, incorporating both scholarly and grey literature, to ensure that a large concourse was derived from a broad range of sources and to maximise the diversity of opinions sampled. With the exception of theses and professional websites, the final Q sample was represented by a relatively similar number of statements (range 4–10) from each document type. This representation was unintentional, and the Delphi panel were unaware of a statement’s specific origin in their decision-making process. It may not have been necessary to conduct a separate search of theses, as the one thesis considered to be most relevant was identified in the scholarly literature search. Professional websites were not particularly useful sources for identifying statements per se, although they identified some relevant linked articles not identified in other searches, from which statements were extracted. Online discussion forums provided many authentic phrases likely to resonate with OTC codeine misusers, representing a potentially underutilized source for obtaining concourse statements for Q studies.

### Q sample size and representation

Similar to the way that R methodology is concerned with ensuring that a representative sample of participants is selected from the target population, in Q methodology the statements forming the Q sample should be representative of the concourse [41]. Stephenson suggests the use of Fisher’s variance design [14] as the most formal way to ensure comprehensiveness of the Q sample, with equal numbers of statements selected from each cell of a theoretically informed two-dimensional matrix. Some Q methodologists, however, advocate for a freer, more creative approach focussing on understanding and representing the statement population as a whole [16]. Fisher’s variance design was not used to structure our Q sample as we were not applying a two-dimensional theory suitable for a matrix design and did not want to force selection of statements to fulfil a predefined quota. Instead, concourse sampling was achieved by thematically grouping and reducing the number of statements using the COM-B model as a theoretical framework, with the final selection of statements decided by the Delphi panel.

The recommended Q sample size of 40–80 statements is based on the balance between providing enough statements to be representative of the concourse while not overtaxing participants [16]. While a number of studies have demonstrated that different Q samples drawn from a single concourse produce similar results [42, 43], further research is required to determine the effect, if any, of Q sample size.

### Use of a theoretical framework

The COM-B model was used to add rigour to the sampling process by providing an evidence-based structure with previous application to addiction research [29]. It was specifically chosen as it is an overarching model incorporating multiple theories of addiction, rather than being based on a single theory. The objective was to reduce the likelihood of analytic bias on identification of themes, to base the themes on existing theory and to lessen the possibility of overlooking theoretically important statements. The COM-B domains and headings provided a useful starting point for the initial sorting of statements, particularly since the concourse was large. However, the COM-B is a broad framework and there was significant overlap between themes, with statements often fitting into more than one of the categories. It was sometimes difficult to decide which category to place statements in. For example, the statements “I use OTC codeine to overcome personal problems” and “I use OTC codeine because circumstances force me to do so” listed in the Opportunity domain under “Cues in the physical and social environment…” could have been placed in “Needs met by the addictive behaviour” in the domain of Motivation. It was also difficult to distinguish between some of the headings such as, “Beliefs about the positive features of the addictive behaviour” and “Pleasure and satisfaction derived from the addictive behaviour”.

Potentially the statements may have been grouped more definitively according to the temporal features of addiction, such as using concepts that describe the addiction life cycle; (1) initial enactment of the behaviour, (2) development of addiction, (3) attempts at recovery or mitigation and (4) relapse [29]. However, this approach may not have adequately represented the multiple theories of addiction, highlighting the importance of careful consideration of the choice and purpose of the theoretical framework. Overall, despite difficulties in allocating statements using the COM-B model, the statements did fit into one or more of the domains and it provided a useful framework to ensure coverage of the major theoretical aspects of addiction.

### Delphi panel size and membership

The final decision on the statements to include in this Q sample was achieved using a Delphi technique with a multidisciplinary panel of addiction experts. Use of this technique aimed to reduce researcher bias in the selection of statements, with decisions being made collectively by experienced addiction experts representing a broad range of disciplines. The Delphi panel also helped to validate the content, representativeness and language of the Q sample. Experts also had the opportunity to comment on and contribute statements that they felt could be important to include.

There is no guiding rule about the number of members required for a Delphi panel [31]. The literature suggests that the size of a panel can range from eight to thousands of participants, with samples on the lower end of the range considered to be acceptable for homogenous panels [31]. Our Delphi panel could be considered to be relatively homogenous, with all members having specific knowledge about OTC codeine dependence. A small, fifteen member panel was therefore recruited, which is similar in size to many other health-related Delphi studies [44–48].

Four of the fifteen experts did not complete Round Two. The time delay of two months between rounds may have contributed to this attrition. Although this response rate of 73% exceeds the suggested 70% requirement to ensure rigour of the Delphi technique [49], a more rapid succession of rounds may have retained the interest of participants and improved retention [44].

Whilst difficult to assess [31], the choice of ‘experts’ to comprise the Delphi panel is based on the requirement that panel members have “knowledge and experience with the issues under investigation” [50]. We chose to consider addiction specialists as experts for our Delphi panel, rather than OTC codeine misusers. The purpose was to obtain a broad, external view of misuser beliefs and to incorporate knowledge of the theories of addiction in the decision making process, rather than focussing on the individual perspectives of misusers. This objective was achieved, as mapping the Q sample against the COM-B confirmed that each of the COM-B domains (and therefore the theories of addiction and the overall concourse) was represented. Codeine misusers themselves also verified that they were able to express their opinions using the Q sample in a subsequent phase of the study.

### Deriving consensus

There are no universally accepted criteria for measuring consensus in Delphi studies [34, 51–53]. Percent agreement, measures of dispersion and stability of responses have each been applied as measures of panel member agreement using a variety of different cut-offs. Delphi studies also quantify the level of agreement with each individual statement. This is usually reported using the median score, rather than the mean, due to the level of measurement used (Likert-type scales are often categorical rather than continuous) and the results may not follow a normal distribution [34].

An interquartile range of less than or equal to one was chosen as the measure of panel consensus for our study on the basis that “IQR of 1 or less is found to be a suitable consensus indicator for 4- or 5- unit scales” [34]. However, a number of researcher reports [34, 38, 39] have made this claim based on the precedence of Raskin [54] and Rayens and Hayn [55], who actually use an interquartile *deviation* (IQD) of ≤1 as their measure of consensus as opposed to IQR ≤ 1. In addition, neither Raskin or Rayens and Hayn reported use of a 5-point scale. Paradoxically, the use of IQR is a more stringent requirement for consensus than IQD, as IQD is half the value of IQR. Other researchers [35, 37] have referenced Linstone and Turoff [32] when suggesting an IQR of 1 to be a good indicator of consensus for 5-point Likert scales. However, this primary source only mentions an “IQR no larger than 2 units on a 10 point scale” [32]. Despite these inconsistencies being identified in the literature, the use of IQR ≤ 1, in combination with the pragmatically chosen median cutoff of ≥4 was adopted for the determination of consensus for our study.

The number of rounds required for a Delphi study is not prescribed. Some researchers set the number of rounds in advance and others continue until the desired level of consensus is achieved [44]. Our Delphi study ceased after two rounds on the basis that consensus on 40–80 statements had been achieved and that the resultant Q sample was representative of the COM-B domains. Had appropriate COM-B representation not occurred, additional Delphi round(s) would have been undertaken. Alternative statements would have been selected from the remaining concourse to represent the missing COM-B domain(s). These new statements would have been presented to the panel using the same consensus criteria for statement inclusion as applied in previous rounds.

### Language issues

In traditional survey design, the wording of questions should be closely aligned to the participants’ usual language to maximise comprehensibility [56]. The same principle applies to the wording of Q sample statements [14]. Modification may therefore be required, for example to simplify, clarify, or avoid the possibility of causing offense [56], particularly if the statements are not sourced directly from potential participants.

In this study, the decision was made to reword statements where possible to remove the words ‘addict’ and ‘addiction’, as panel members suggested that these terms could potentially stigmatise codeine misusers. This potential for stigmatisation was supported by existing literature [57, 58]. The choice of replacement words was difficult due to a lack of consistency in addiction diagnostic terminology and the changing nature and continued debate around the lexicon of addiction [59]. ‘Dependence’, as used by The International Classification of Diseases [60], was ultimately chosen as the most suitable replacement word over ‘substance use disorder’, as used by the Diagnostic and Statistical Manual of Mental Disorders [61], as the former implies compulsive use and is more concise. However, this was not done without recognising its limitations, as many of the statements were direct quotes from codeine misusers who referred to themselves as ‘addicts’. This suggested that the term may be a normal part of their vernacular and potentially a suitable choice for a survey attempting to use the language of the participants. In addition, the word dependence has a dual meaning, traditionally referring to the normal physiological adaptations that occur in response to repeated drug administration rather than being associated with compulsive use [62].

A limitation of this study is that the language used was not validated by codeine misusers prior to finalising the Q sample. The statements could potentially have been piloted with codeine misusers after completion of the Delphi component, however limited access to potential participants precluded this option.

The Delphi panel were provided with written information outlining the task, including the background of the study, the aim and instructions. However, three participants asked for further explanation and clarification about whether their responses should reflect their personal views of dependence or the views likely to be expressed by misusers. This potential ambiguity may have affected the reliability of the panel responses and highlights the importance of providing clear and specific instructions, particularly when using a methodology that participants may be unfamiliar with. In addition, the majority of experts had knowledge of and experience with other types of misusers as well as OTC codeine misusers. This may have led to the inclusion of some views of dependence not specific to OTC codeine. Despite these limitations, the Delphi technique was successfully incorporated into the process of Q sample construction as a mechanism to reduce researcher bias and produce a Q sample suited to codeine misusers.

## Conclusion

This paper explicitly describes and discusses a novel and rigorous approach to Q sample construction involving the successful incorporation of a literature review, use of a theoretical framework and a Delphi technique with a panel of experts. Methodological issues were critically examined, including the importance of reducing researcher bias, justifying and accurately reporting decisions made during the research process and exercising due diligence when basing decisions on precedence. Further research is recommended to clarify the optimal number of statements for the Q sample, the size and composition of a Delphi panel, the definition of Delphi consensus and to confirm the Delphi technique as a useful method for concourse reduction. This new approach to Q sample construction could be useful for those considering Q methodology and for furthering the rigour of this research technique.

## Abbreviations

COM-B: Capability, opportunity, motivation – behaviour
IQD: Interquartile deviation (or quartile deviation)
IQR: Interquartile range
OTC: Over-the-counter

## References

[1] Flurey CA, Morris M, Pollock J, Richards P, Hughes R, Hewlett S (2014). A Q-methodology study of flare help-seeking behaviours and different experiences of daily life in rheumatoid arthritis. BMC Musculoskelet Disord.

[2] Patty NJ, van Dijk HM, Wallenburg I, Bal R, Helmerhorst TJ, Van Exel J (2017). To vaccinate or not to vaccinate? Perspectives on HPV vaccination among girls, boys, and parents in the Netherlands: a Q-methodological study. BMC Public Health.

[3] Berghout M, van Exel J, Leensvaart L, Cramm JM (2015). Healthcare professionals’ views on patient-centered care in hospitals. BMC Health Serv Res.

[4] Shabila NP, Al-Tawil NG, Al-Hadithi TS, Sondorp E (2014). Using Q-methodology to explore people’s health seeking behavior and perception of the quality of primary care services. BMC Public Health.

[5] Killam LA, Montgomery P, Raymond JM, Mossey S, Timmermans KE, Binette J (2012). Unsafe clinical practices as perceived by final year baccalaureate nursing students: Q methodology. BMC Nurs.

[6] Hazen AC, Van Der Wal AW, Sloeserwij VM, Zwart DL, De Gier JJ, De Wit NJ (2016). Controversy and consensus on a clinical pharmacist in primary care in the Netherlands. Int J Clin Pharm.

[7] Alderson S, Foy R, Bryant L, Ahmed S, House A (2018). Using Q-methodology to guide the implementation of new healthcare policies. BMJ Qual Saf.

[8] van Exel J, Baker R, Mason H, Donaldson C, Brouwer W (2015). Public views on principles for health care priority setting: findings of a European cross-country study using Q methodology. Soc Sci Med.

[9] Baker R, Wildman J, Mason H, Donaldson C (2014). Q-ing for health - a new approach to eliciting the public’s views on health care resource allocation. Health Econ.

[10] Waterval Dominique G. J., Frambach Janneke M., Driessen Erik W., Muijtjens Arno, Scherpbier Albert J. J. A. (2018). Connected, attracted, and concerned: A Q study on medical crossborder curriculum partnerships. Medical Teacher.

[11] Fokkema JP, Scheele F, Westerman M, van Exel J, Scherpbier AJ, van der Vleuten CP (2014). Perceived effects of innovations in postgraduate medical education: a Q study focusing on workplace-based assessment. Acad Med.

[12] Stephenson W (1935). Technique of factor analysis. Nature..

[13] Stephenson W (1935). Correlating persons instead of tests. J Pers.

[14] Brown SR (1980). Political subjectivity: applications of Q methodology in policial science.

[15] Burt C, Stephenson W (1939). Alternative views on correlations between persons. Psychometrika..

[16] Watts S, Stenner P (2012). Doing Q methodological research: theory, method and interpretation.

[17] Cross RM (2005). Exploring attitudes: the case for Q methodology. Health Educ Res.

[18] Kampen JK, Tamás P (2014). Overly ambitious: contributions and current status of Q methodology. Qual Quant.

[19] Block J. The Q-sort in character appraisal: encoding subjective impressions of persons quantitatively: American Psychological Association; 2008.

[20] Fontein-Kuipers Y (2016). Development of a Q-set for a Q-method study about midwives’ perspectives of woman-centered care. Health Edu Care.

[21] Paige JB, Morin KH (2016). Q-sample construction: a critical step for a Q-methodological study. West J Nurs Res.

[22] Kenward Linda (2019). A literature review to guide novice researchers using Q methodology in the development of a framework for concourse management. Nurse Researcher.

[23] Wallis J, Burns J, Capdevila R (2009). Q methodology and a Delphi poll: a useful approach to researching a narrative approach to therapy. Qual Res Psychol.

[24] Rust NA (2017). Can stakeholders agree on how to reduce human–carnivore conflict on Namibian livestock farms? A novel Q-methodology and Delphi exercise. Oryx..

[25] Michie S, Van Stralen MM, West R (2011). The behaviour change wheel: a new method for characterising and designing behaviour change interventions. Implement Sci.

[26] Sinnott C, Mercer SW, Payne RA, Duerden M, Bradley CP, Byrne M (2015). Improving medication management in multimorbidity: development of the MultimorbiditY COllaborative medication review and DEcision making (MY COMRADE) intervention using the behaviour change wheel. Implement Sci.

[27] Gardner B, Smith L, Lorencatto F, Hamer M, Biddle SJ (2016). How to reduce sitting time? A review of behaviour change strategies used in sedentary behaviour reduction interventions among adults. Health Psychol Rev.

[28] Webb J, Foster J, Poulter E (2016). Increasing the frequency of physical activity very brief advice for cancer patients. Development of an intervention using the behaviour change wheel. Public Health.

[29] West R. Models of Addiction. EMCDDA Insight Series No14. Luxembourg: European monitoring Centre for drugs and drug addiction; 2013.

[30] Dalkey N, Helmer O (1963). An experimental application of the Delphi method to the use of experts. Manag Sci.

[31] Keeney S, McKenna H, Hasson F (2011). The Delphi technique in nursing and health research.

[32] Linstone HA, Turoff M (1975). The Delphi method: techniques and applications.

[33] LimeSurvey GH. LimeSurvey: an open source survey tool. LimeSurvey GmbH. Hamburg. http://www.limesurvey.org. Accessed 28 October 2018.

[34] von der Gracht HA (2012). Consensus measurement in Delphi studies. Technol Forecast Soc Change.

[35] O’Donovan A, Mohile S, Leech M (2015). Expert consensus panel guidelines on geriatric assessment in oncology. Eur J Cancer Care.

[36] Jünger S, Payne S, Brearley S, Ploenes V, Radbruch L (2012). Consensus building in palliative care: a Europe-wide Delphi study on common understandings and conceptual differences. J Pain Symptom Manag.

[37] Vandelanotte C, Dwyer T, Van Itallie A, Hanley C, Mummery WK (2010). The development of an internet-based outpatient cardiac rehabilitation intervention: a Delphi study. BMC Cardiovasc Disord.

[38] Fuermaier A, Fricke J, deVries S, Tucha L, Tucha O. Neuropsychological assessment of adults with ADHD: a Delphi consensus study. Appl Neuropsych-Adul. 2018. 10.1080/23279095.2018.1429441. 10.1080/23279095.2018.1429441 29424567

[39] McMahon S, Cusack T, O’Donoghue G (2014). Barriers and facilitators to providing undergraduate physiotherapy clinical education in the primary care setting: a three-round Delphi study. Physiotherapy..

[40] Smith J, Noble H (2014). Bias in research. Evid Based Nurs.

[41] Baker R, Thompson C, Mannion R (2006). Q methodology in health economics. J Health Serv Res Policy.

[42] Hilden AH (1958). Q-sort correlation: stability and random choice of statements. J Consult Psychol.

[43] Daily JH (1973). Dimensions of political attitudes: a Q technique study of public reactions to the Calley verdict [dissertation]. Kent. Ohio: Kent State University.

[44] Trevelyan EG, Robinson N (2015). Delphi methodology in health research: how to do it?. Eur J Integr Med.

[45] Dolan C, Glynn R, Lawlor B (2017). A Delphi study to establish an expert consensus opinion on risk factors for type 2 diabetes, and potential complications of diabetes, including brain health associations. Eur Psychiatry.

[46] Davies E, Martin J, Foxcroft D (2016). Development of an adolescent alcohol misuse intervention based on the prototype willingness model: a Delphi study. Health Educ.

[47] Primdahl SC, Todsen T, Clemmesen L, Knudsen L, Weile J (2016). Rating scale for the assessment of competence in ultrasound-guided peripheral vascular access–a Delphi consensus study. J Vasc Access.

[48] Maverakis E, Ma C, Shinkai K, Fiorentino D, Callen JP, Wollina U (2018). Diagnostic criteria of ulcerative pyoderma gangrenosum: a Delphi consensus of international experts. JAMA dermatology.

[49] Sumsion T (1998). The Delphi technique: an adaptive research tool. Br J Occup Ther.

[50] Adler M, Ziglio E (1996). Gazing into the oracle: the Delphi method and its application to social policy and public health.

[51] Giannarou L, Zervas E (2014). Using Delphi technique to build consensus in practice. Int J Bus Sci Appl Manag.

[52] Diamond IR, Grant RC, Feldman BM, Moore AM, Wales PW, Pencharz PB (2014). Defining consensus: a systematic review recommends methodologic criteria for reporting of Delphi studies. J Clin Epidemiol.

[53] Holey EA, Feeley JL, Dixon J, Whittaker VJ (2007). An exploration of the use of simple statistics to measure consensus and stability in Delphi studies. BMC Med Res Methodol.

[54] Raskin MS (1994). The Delphi study in field instruction revisited: expert consensus on issues and research priorities. J Soc Work Educ.

[55] Rayens MK, Hahn EJ (2000). Building consensus using the policy Delphi method. Policy Polit Nurs Pract.

[56] Robinson SB, Leonard KF (2019). Designing quality survey questions.

[57] Kelly J, Wakeman S, Saitz R (2015). Stop talking ‘dirty': clinicians, language, and quality of care for the leading cause of preventable death in the United States. Am J Med.

[58] Broyles LM, Binswanger IA, Jenkins JA, Finnell DS, Faseru B, Cavaiola A (2014). Confronting inadvertent stigma and pejorative language in addiction scholarship: a recognition and response. Subst Abus.

[59] Kelly JF, Saitz R, Wakeman S (2016). Language, substance use disorders, and policy: the need to reach consensus on an “addiction-ary”. Alcohol Treat Q.

[60] World Health Organization. The international classification of diseases 11th revision browser, Geneva. 2018. https://icd.who.int/browse11/l-m/en. Accessed 28 October 2018.

[61] American Psychiatric Association (2013). Diagnostic and statistical manual of mental disorders.

[62] O'Brien C (2011). Addiction and dependence in DSM-V. Addiction..

